# Ten-Year Follow-Up of a Randomized Clinical Trial of Total Thyroidectomy Versus Dunhill Operation Versus Bilateral Subtotal Thyroidectomy for Multinodular Non-toxic Goiter

**DOI:** 10.1007/s00268-017-4230-1

**Published:** 2017-09-23

**Authors:** Marcin Barczyński, Aleksander Konturek, Alicja Hubalewska-Dydejczyk, Filip Gołkowski, Wojciech Nowak

**Affiliations:** 1 Department of Endocrine Surgery, Third Chair of General Surgery, Faculty of Medicine, Jagiellonian University Medical College, 37 Prądnicka Street, 31-202 Kraków, Poland; 2 Chair and Department of Endocrinology, Faculty of Medicine, Jagiellonian University Medical College, Kraków, Poland; 3 Department of Endocrinology and Internal Medicine, Faculty of Medicine, Andrzej Frycz Modrzewski Krakow University, Kraków, Poland

## Abstract

**Background:**

The aim of this study was to validate in a 10-year follow-up the initial outcomes of various thyroid resection methods for multinodular non-toxic goiter (MNG) reported in World J Surg 2010;34:1203–13.

**Materials and methods:**

Six hundred consenting patients with MNG were randomized to three groups of 200 patients each: total thyroidectomy (TT), Dunhill operation (DO), bilateral subtotal thyroidectomy (BST). Obligatory follow-up period of 60 months was extended up to 120 months for all the consenting patients. The primary outcome measure was the prevalence of recurrent goiter and need for revision thyroid surgery. The secondary outcome measure was the cumulative postoperative and post-revision morbidity rate.

**Results:**

The primary outcomes were twice as inferior at 10 years when compared to 5-year results for DO and BST, but not for TT. Recurrent goiter was found at 10 years in 1 (0.6%) TT versus 15 (8.6%) DO versus 39 (22.4%) BST (*p* < 0.001), and revision thyroidectomy was necessary in 1 (0.6%) TT versus 5 (2.8%) DO versus 14 (8.0%) BST patients (*p* < 0.001). Any permanent morbidity at 10 years was present in 5 (2.8%) TT patients following initial surgery versus 7 (4.0%) DO and 10 (5.7%) BST patients following initial and revision thyroidectomy (nonsignificant differences). At 10 years, 23 (11.5%) TT versus 25 (12.5%) DO versus 26 (13.0%) BST patients were lost to follow-up.

**Conclusions:**

Total thyroidectomy can be considered the preferred surgical approach for patients with MNG, as it abolishes the risk of goiter recurrence and need for future revision thyroidectomy when compared to more limited thyroid resections, whereas the prevalence of permanent morbidity is not increased at experienced hands.

**Registration number:**

NCT00946894 (http://www.clinicaltrials.gov).

## Introduction

In recent years, total thyroidectomy has become increasingly popular in the treatment of bilateral multinodular non-toxic goiter (MNG), replacing subtotal thyroidectomy in many high-volume endocrine surgery units worldwide [1, 2]. Nevertheless, the major benefits of total thyroidectomy understood as the risk of recurrent goiter reduced to almost zero and abolished need for revision thyroid surgery in the future should be balanced against the risk of postoperative morbidity. Thus, total thyroidectomy for benign thyroid disease continues to remain controversial, as there are many conflicting data published in the literature regarding the risk of hypoparathyroidism and recurrent laryngeal nerve injury stratified to indications for surgery, extent of thyroid resection and surgical volume [2–5]. Cirocchi et al. [6] published recently a Cochrane systematic review focused on total and near-total thyroidectomy versus subtotal thyroidectomy for MNG in adults and identified only four randomized controlled trials (RCT) in the field. Despite the fact that goiter recurrence was found to be reduced following total thyroidectomy (TT), the effects on other major endpoints, such as the need for revision surgery for goiter recurrence, prevalence of adverse events, and thyroid cancer incidence, remained unclear. Hence, new RCTs with a long-term follow-up and with additional focus on data such as surgical experience, surgical volume, and more attention to surgical technique were found to be needed [6]. To fulfill this gap in evidence with more data, it was decided at our institution to continue follow-up of all consenting patients previously included into RCT for 5 years [7]. Thus, the aim of this study was to validate in a 10-year follow-up the outcomes of RCT run at our institution and published in 2010 comparing results of various thyroid resection modes hitherto assessed within 5 years following surgery [7]. The hypothesis explored at the present study was that the prevalence of recurrent goiter and need for revision thyroidectomy would increase with time of follow-up and that the cumulative risk of postoperative and post-revision morbidity of more limited thyroid resection modes would overweight the initial risk of total thyroidectomy.

## Materials and methods

### Study design and patient selection

Patients referred to the Department of Endocrine Surgery, Third Department of General Surgery, Jagiellonian University Medical College in Krakow, for first-time thyroid surgery between January 2000 and December 2003 were registered. Eligible patients with MNG were assessed for the study. The study was approved by the institutional review board.

The inclusion criterion was a bilateral non-toxic MNG with the posterior aspects of both thyroid lobes appearing normal on ultrasound of the neck.

The exclusion criteria included: MNG involving the posterior aspect/s of thyroid lobe/s, suspicion of thyroid cancer, previous thyroid surgery, thyroiditis, subclinical or clinically overt hypothyroidism or hyperthyroidism, pregnancy or lactation, age < 18 years or > 65 years, ASA 4 grade (American Society of Anesthesiology), and inability to comply with the follow-up protocol.

Patients who signed the informed consent were randomized to three groups: total thyroidectomy (TT), Dunhill operation (DO), and bilateral subtotal thyroidectomy (BST).

The primary outcome measure was the prevalence of recurrent goiter and need for redo surgery. The secondary outcome measure was the cumulative postoperative and post-revision morbidity rate (hypoparathyroidism and recurrent laryngeal nerve injury).

### Randomization

The randomization sequence was generated by a computer. Sequencing was based on permuted blocks of two and three to balance the number of patients in the treatment groups. The patients were allocated randomly to one of the three treatment groups in a 1:1:1 ratio. Information on the type of intervention remained in consecutively numbered and sealed envelopes that were stored in the operating theater. An envelope containing the allocation was added to the patient’s file in the operating room. The envelope was opened, and the surgeon performed the assigned intervention. All the participants were blinded to treatment assignment for the duration of the study.

### Anesthesia

Operations in both groups were performed under general anesthesia. Two anesthesiologists involved in the study followed a strict protocol including premedication with IV midazolam and anesthesia induction with fentanyl, thiopental, and pancuronium at the body mass-dependent dose. After the endotracheal intubation, all the patients were put on mechanical ventilation (sevoflurane and oxygen mixture).

### Surgical technique

All the operations were performed by one of the three experienced endocrine surgeons involved in the study. Each of them performed approximately one-third of the operations in each study arm. In the TT group, the operation consisted of extracapsular total thyroidectomy, and in the DO group, the operation consisted of unilateral extracapsular total thyroidectomy and contralateral subtotal thyroid lobe resection (leaving a thyroid stump of approximately 2 g of normal remnant tissue), whereas in the ST group, the operation consisted of bilateral subtotal thyroidectomy (leaving thyroid stumps of approximately 2 g of normal remnant tissue each on both sides of the neck). In each group, efforts were made to identify and remove the entire pyramidal thyroid lobe. In each patient, the recurrent laryngeal nerves were exposed and the branches of the superior and inferior thyroid arteries were divided close to the thyroid capsule (peripheral ligation). Intraoperative nerve monitoring (IONM) was not used for initial surgery in this study. However, IONM was utilized for 15 of 20 (75%) revision operations for recurrent goiter in this study, depending on individual surgical preferences. A lateral approach was routinely used for all reoperative cases. The surgical technique of reoperation for recurrent goiter was described in detail in our previous publication [8]. The parathyroid glands were meticulously dissected from the thyroid gland, and effort was made to identify all the four parathyroid glands and preserve as many as possible “in situ.” Any inadvertently removed parathyroid gland found on inspection to be lying on the thyroid capsule, any gland that was anatomically impossible to be preserved, as well as any devascularized gland were electively reimplanted into the sternocleidomastoid muscle using the standard technique of parathyroid autotransplantation as described by Wells et al. [9].

### Preoperative evaluation and postoperative follow-up

High-resolution Doppler ultrasound of the neck with both 7.5- and 12-MHz linear array transducers (Logiq 7; GE, Solingen, Germany) was performed during an outpatient visit prior to admission by a single endocrine surgeon (MB) experienced in thyroid ultrasound imaging. Thyroid volumes were calculated according to the spherical ellipsoid formula: volume = *π*/6 × anteroposterior diameter (cm) × width (cm) × length (cm). Fine-needle aspiration (FNA) was performed in all the patients prior to enrollment. Preoperative evaluation included serum free T3, free T4, thyroid-stimulating hormone (TSH) concentrations (respectively by commercial radioimmunoassay kits and ultrasensitive method) and serum thyroid peroxidase antibodies (TPOAb) levels.

All the patients underwent ultrasonographic, cytological, and biochemical follow-up for 60 months postoperatively. However, for all the consenting patients, the follow-up period was extended to 120 months postoperatively. All the patients were evaluated at 3, 6, 9, and 12 months postoperatively during the first year, and every 12 months for the following years. Biochemical evaluation consisted of determining serum TSH concentrations. Thyroid ultrasonography was performed by the same operator (MB) using the same equipment as in preoperative evaluation (Logiq 7; GE, Solingen, Germany). All the patients in this study, irrespectively of the individual group assignment, received postoperative levothyroxine treatment. The levothyroxine dose was adjusted to serum TSH concentrations to keep it within the lowest two-thirds of the reference range (0.3–2.5 mU/L). This therapeutic strategy was focused on avoiding the risk of mild thyrotoxicosis and limiting the excessive TSH stimulation of the thyroid remnants (in the DO and BST groups).

The following criteria were used to define recurrence of nodular lesions within the remnant thyroid tissue (the same as previously reported in the Miccoli study): presence of hypoechoic or hyperechoic nodular pattern at least 5 mm in diameter, identification of perinodular hypoechogenic or hyperechogenic halo, and presence of an anechoic lesion with a reinforced posterior wall [10]. FNA was electively performed during the follow-up period in all the cases of identified thyroid lesions larger than 1 cm in diameter within the remnant thyroid tissue. The following indications for reoperation were used: presence of a 3 cm, or larger nodule, result of FNA suggestive of an increased risk for malignancy, and presence of compressive symptoms. Indirect laryngoscopy by an ENT specialist was mandatory before surgery and before discharge. In patients with RLN paresis, an additional examination was scheduled at 2 weeks and 1, 2, 4, 6, and 12 months after surgery, or until the vocal cord function recovered. Vocal cord paresis for more than 12 months after the operation was regarded as permanent palsy.

The patients were monitored for postoperative biochemical hypocalcemia at 12, 24, 48, and 72 h postoperatively (during hospitalization and after discharge on morning outpatient visits), with hypocalcemia being defined as a total serum calcium level lower than 2.0 mmol/L, in either asymptomatic or symptomatic patients. Persistent hypocalcemia for more than 6 months after the operation was regarded as permanent hypoparathyroidism.

### Statistical analysis

The sample size was estimated based on the principle of detecting a 5% difference in the prevalence of recurrent goiter with a 90% probability at *p* < 0.05. The univariate relation between patient characteristics and the development of goiter recurrence and the need for reoperation were examined. The statistical significance of categorical variables was evaluated by the *χ* ^2^ test and *F* test, whereas the Student’s *t*-test was used to evaluate continuous variables. Ten-year recurrence-free survival was calculated using the Kaplan–Meier method, with the log rank test for comparison between study groups. All the data were entered onto a dedicated spreadsheet (Microsoft Excel 2010; Microsoft Corporation, San Jose, CA, USA) by a medical assistant and then analyzed by a statistician (MedCalc, version 16, Belgium). *p* < 0.05 was considered to indicate significance.

## Results

**Fig. 1:**
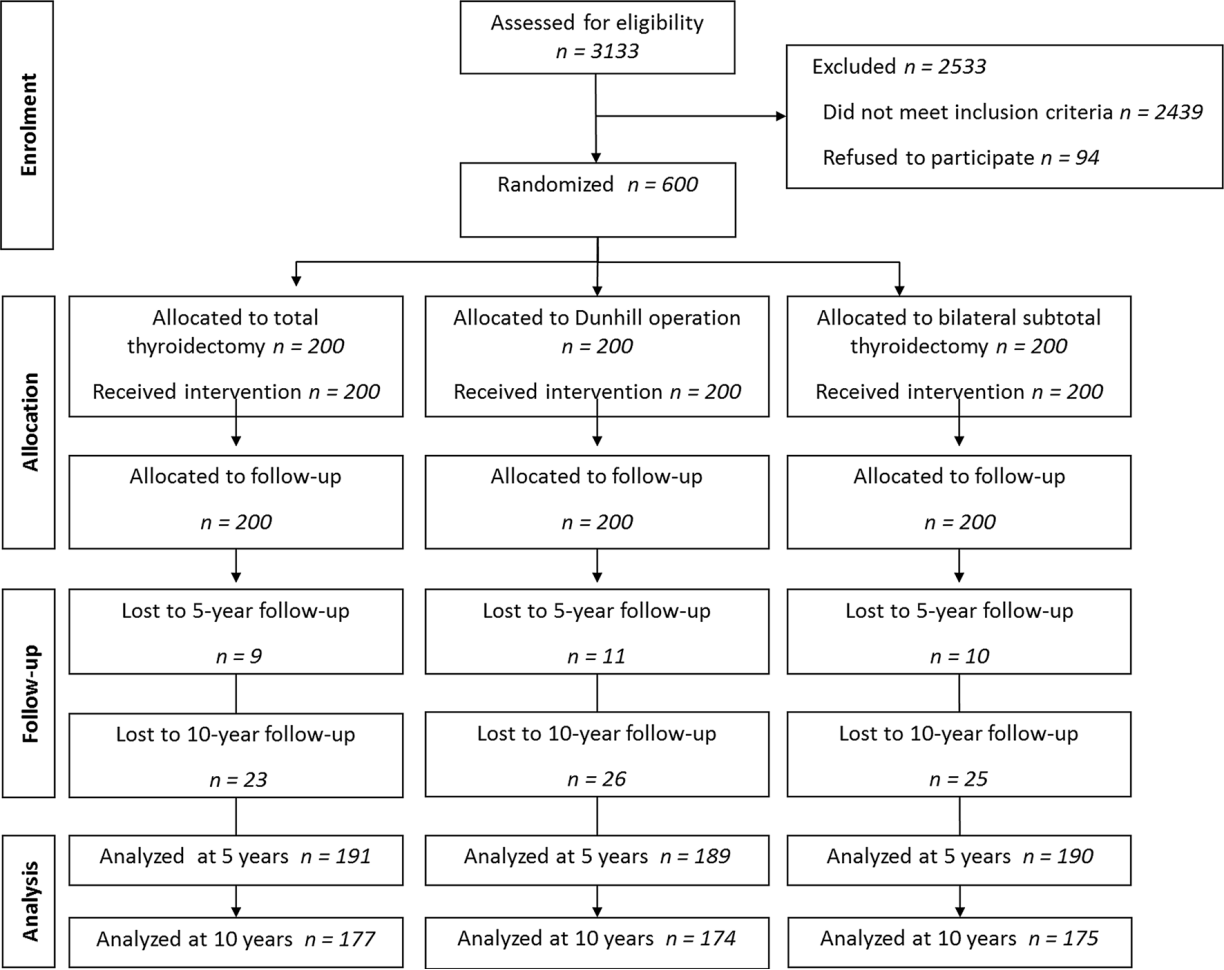
Flow diagram of the study

Three thousands one hundred and thirty-three patients were referred to the Department of Endocrine Surgery, Third Chair of General Surgery, Jagiellonian University College of Medicine in Krakow, Poland, for first-time thyroid surgery between January 2000 and December 2003. Of this group, 694 patients were eligible for this study, while 94 patients refused to participate. Finally, 600 patients who signed the informed consent were randomized to three groups equal in size (*n* = 200): TT, DO, and BST. Thirty patients were lost to follow-up at 5 years, leaving 570 who were followed for at least 60 months: 191 in the TT group, 189 in the DO group, and 190 in the BST group. Further 44 patients were lost to follow-up at 10 years, leaving 526 who were followed for at least 120 months: 177 in the TT group, 174 in the DO group, and 175 in the BST group (Fig. 1). Demographic characteristics of patients in this study are shown in Table 1. In this study, indications for initial surgery for MNG were: presence of compressive symptoms in a patient with a thyroid nodule/s of 3 cm or larger (*n* = 412), indeterminate result of FNA suggestive of an increased risk for malignancy (*n* = 91), discrepancy between the suspicious ultrasound phenotype of the dominant thyroid lesion/s, and the FNA result suggestive for non-diagnostic or benign lesion (*n* = 45), particularly in cases of a cold thyroid scanning, multiple bilateral thyroid lesions not responding to TSH suppressive treatment with l-thyroxine (*n* = 49), and on patient’s request for cosmetic indications (*n* = 3). All indications were equally distributed between the three study arms.

**Table 1:** Demographic characteristics of 526 patients who completed the 10-year follow-up

	TT *n* = 177	DO *n* = 174	BST *n* = 175
Sex ratio^a^ (M:F)	15:162	18:156	15:160
Mean age^b^ (years)	45.9 ± 13.9	47.0 ± 15.3	47.9 ± 15.2
Preoperative TSH^b^ (mIU/L)	1.87 ± 0.84	1.82 ± 0.81	1.80 ± 0.91
Preoperative thyroid volume (assessed by ultrasound)^a^ (mL)	75.8 ± 38.4	77.8 ± 39.5	78.8 ± 39.9

*TT* total thyroidectomy, *DO* Dunhill operation, *BST* bilateral subtotal thyroidectomy

^a^ *χ* ^2^ test

^b^ *t*-test; statistically nonsignificant differences for all values

### Primary endpoints

**Fig. 2:**
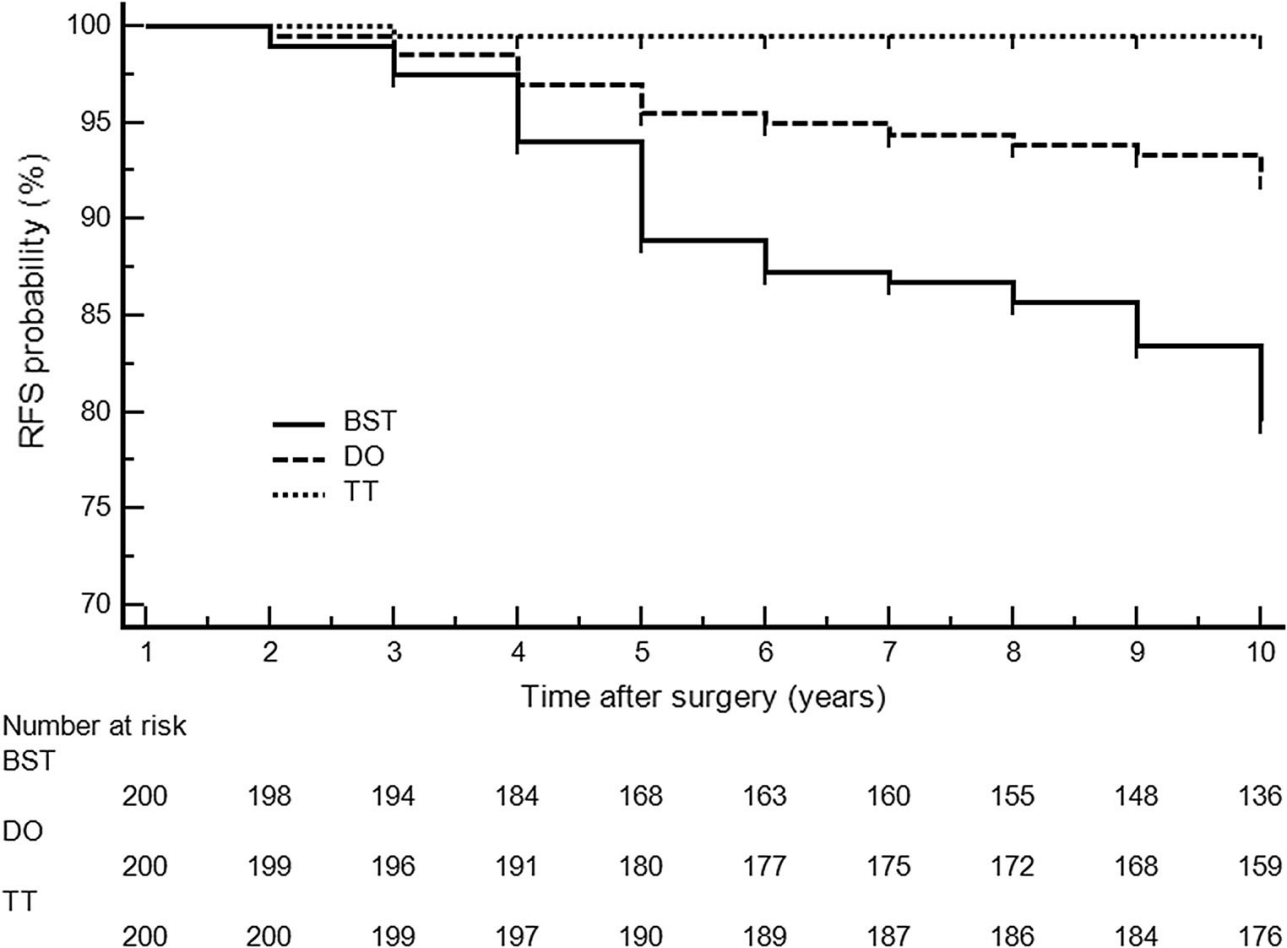
Recurrence free survival (RFS) for the cohort of 600 patients treated for multinodular non-toxic goiter by total thyroidectomy (TT), Dunhill operation (DO), and bilateral subtotal thyroidectomy (BST). TT significantly decreased the risk of recurrence when compared to BST: HR 0.795 (0.643–0.982), *p* < 0.001 at 10 years (log rank test)

Recurrent goiter was found at 10 years in 1 (0.6%) TT versus 15 (8.6%) DO versus 39 (22.4%) BST patients (*p* < 0.001 for TT vs. DO or BST and for DO vs. BST), and revision thyroidectomy was necessary in 1 (0.6%) TT versus 5 (2.8%) DO versus 14 (8.0%) BST subjects (*p* < 0.001 for TT vs. BST and *p* = 0.019 for DO vs. BST). Detailed data are shown in Table 2. Recurrence-free survival (RFS) for the cohort of 600 patients treated for multinodular non-toxic goiter by total thyroidectomy (TT), Dunhill operation (DO), and bilateral subtotal thyroidectomy (BST) is shown in Fig. 2. TT significantly decreased the risk of recurrence when compared to BST: HR 0.795 (0.643–0.982), *p* < 0.001 at 10 years (log rank test).

**Table 2:** Prevalence of recurrent goiter and need for revision thyroidectomy for 526 patients who completed the 10-year follow-up

	TT	P TT versus DO	DO	P DO versus BST	BST	P TT versus BST
Lost to follow-up at 10 years	23 (11.5)	0.296	26 (13.0)	0.329	25 (12.5)	0.343
Recurrent goiter [no (%)]	1 (0.6)	**<0.001**	15 (8.6)	**<0.001**	39 (22.3)	**<0.001**
Need for revision thyroidectomy [no (%)]	1 (0.6)	0.070	5 (2.9)	**0.019**	14 (8.0)	**<0.001**

Bold values are statistically significant (*p* < 0.05)

*TT* total thyroidectomy, *DO* Dunhill operation, *BST* bilateral subtotal thyroidectomy

*χ* ^2^ test for all

### Secondary endpoints

Cumulative permanent postoperative hypoparathyroidism after initial surgery and revision thyroidectomy was present in 1 (0.6%) TT versus 2 (1.1%) DO versus 5 (2.9%) BST patients (nonsignificant differences), whereas the cumulative permanent recurrent laryngeal nerve injury was found in 4 (1.1%) TT versus 5 (1.4%) DO versus 5 (1.4%) BST nerves at risk (nonsignificant differences). Detailed data are shown in Table 3. The primary outcomes were twice as inferior at 10 years when compared to 5-year results for DO and BST, but not for TT [7]. No significant differences were observed between the three surgeons involved in this study and the outcomes of surgery they performed.

**Table 3:** Complications after initial versus revision thyroidectomy and cumulative risk of morbidity among 526 patients who completed the 10-year follow-up

	TT	P TT versus DO	DO	P DO versus BST	BST	P TT versus BST
*Risk of initial thyroid surgery (n* *=* *600)* ^*a*^						
*n*	200		200		200	
Hypoparathyroidism [no (%)]						
Total	22 (11.5)	**0.007**	8 (4.2)	0.241	4 (2.1)	**<0.001**
Transient	21 (11.0)	**0.012**	8 (4.2)	0.241	4 (2.1)	**<0.001**
Permanent	1 (0.5)	0.316	0 (0)	1.000	0 (0)	0.316
Recurrent laryngeal nerve injury [no (%)]						
Total	25 (6.5)	0.352	19 (5.0)	0.088	10 (2.6)	**0.009**
Temporary	21 (5.5)	0.399	16 (4.2)	0.097	8 (2.1)	**0.014**
Permanent	4 (1.0)	0.704	3 (0.8)	0.653	2 (0.5)	0.412
Hemorrhage [no (%)]	0 (0)	0.316	1 (0.5)	0.562	2 (1.0)	0.156
*Risk of revision thyroidectomy (n* *=* *20)* ^*b*^						
*n*	1		5		14	
Hypoparathyroidism [no (%)]						
Total	0 (0)	1.000	3 (60.0)	1.000	10 (71.4)	0.333
Transient	0 (0)	1.000	1 (10.0)	1.000	5 (35.7)	1.000
Permanent	0 (0)	1.000	2 (40.0)	1.000	5 (35.7)	1.000
Recurrent laryngeal nerve injury [no (%)]						
Total	0 (0)	1.000	3 (60.0)	0.411	5 (17.8)	1.000
Temporary	0 (0)	1.000	1 (20.0)	1.000	2 (7.1)	1.000
Permanent	0 (0)	1.000	2 (40.0)	0.592	3 (10.7)	1.000
Hemorrhage [no (%)]	0 (0)	1.000	0 (0)	1.000	1 (7.1)	1.000
*Cumulative risk of initial and revision thyroidectomy (n* *=* *526)* ^*a*^						
Permanent hypoparathyroidism [no (%)]	1 (0.6)	0.555	2 (1.1)	0.252	5 (2.9)	0.097
Permanent recurrent laryngeal nerve injury [no (%)]	4 (1.1)	0.717	5 (1.4)	1.000	5 (1.4)	0.724
Hemorrhage [no (%)]	1 (0.6)	1.000	1 (0.6)	0.317	3 (1.7)	0.309

Bold values are statistically significant (*p* < 0.05)

*TT* total thyroidectomy, *DO* Dunhill operation, *BST* bilateral subtotal thyroidectomy calculation for nerves at risk, not for patients

^a^ *χ* ^2^ test

^b^ *F* test

## Discussion

Total thyroidectomy was proposed as the definitive treatment for MNG in order to reduce the risk of goiter recurrence to almost zero [11, 12]. The evidence regarding the balance between the effectiveness and safety of TT compared with more limited thyroid resection modes, such as DO or BST for MNG, is conflicting and no consensus has been reached [3, 6, 13].

In the present study, the prevalence of recurrent goiter and need for future revision thyroidectomy during 10-year follow-up were significantly reduced after TT as compared to DO and BST (Table 2). Prevalence of goiter recurrence tended to increase with time of follow-up (Fig. 2), and goiter recurrence noted at 5 years (8.2%) was almost doubled at 10 years (15.5%) following the initial surgery being more limited than TT (*p* = 0.002) [7]. In addition, the need for revision thyroidectomy for goiter recurrence in procedures more limited than TT was more than sixfold higher during 10-year follow-up (5.4%) as compared to values noted during 5-year follow-up (0.8%), whereas it remained almost abolished following TT (0.6%) during the entire study period (*p* < 0.001) [7].

On the other hand, the risk of temporary, but not permanent RLN injury, as well as transient, but not permanent hypoparathyroidism, was significantly higher following initial TT as compared to less than total thyroid resections. However, the risk of initial TT did not overweight the cumulative risk of postoperative and post-revision permanent morbidity of more limited thyroid resection modes (Table 3).

Much of the debate regarding the extent of surgical resection in MNG was inspired by the previously reported higher prevalence of permanent morbidity following TT [14–16]. However, more recent data indicate the safety of TT for benign thyroid disease if surgery is performed by high-volume surgeons [2, 5, 7, 11, 12, 17–20], which is also supported by the outcomes of the present study. Patients after procedures more limited than TT are at an increased risk of goiter recurrence and need lifelong surveillance [21]. Subclinical ultrasound-detectable lesions within the thyroid stumps following subtotal thyroidectomy are much more frequent than previously thought, approaching 50% of patients undergoing lifelong follow-up [21]. Some of these ultrasound findings were even the reasons for surgical malpractice claims [22]. However, clinically overt recurrent MNG occurs in up to one-third of patients with ultrasound-detectable lesions, with a peak incidence between 10 and 20 years following the initial procedures more limited than TT [23]. The longer the follow-up period, the higher the prevalence of recurrent goiter. Despite the fact that indications for reoperation for goiter recurrence are rare, the redo surgery may be necessary in approximately 5% of patients following initial subtotal thyroidectomy [3]. Similar observations were made in the present study. However, Delbridge et al. [23] reported much higher values and showed that subtotal thyroidectomy for MNG resulted in reoperation for recurrent disease in up to 20% of patients, reaching a top incidence 13 years following the primary surgery. According to the 2015 ATA management guidelines, surgery may be considered for growing nodules that are benign after repeat FNA if they are large (> 4 cm), causing compressive or structural symptoms, or the indications may be based upon clinical concerns (Recommendation 27 A) [20]. Moreover, patients with indeterminate nodules who have bilateral nodular disease, those with significant medical comorbidities, or those who prefer to undergo bilateral thyroidectomy to avoid the possibility of requiring a future surgery on the contralateral lobe, may undergo total or near-total thyroidectomy, assuming completion thyroidectomy would be recommended if the indeterminate nodule proved malignant following lobectomy (Recommendation 20 B) [20]. In addition, indications for completion thyroidectomy can be expected in one-third of patients with incidentally diagnosed thyroid cancer following BST [3]. As a result, at least one in ten patients following a procedure more limited than TT may require revision thyroidectomy in the future. It has been repeatedly shown that the risk of repeat thyroid surgery is up to 20-fold higher as compared to the risk of initial TT [3, 4, 23]. Identification and preservation of vital anatomic structures, such as recurrent laryngeal nerves and parathyroid glands, may be compromised during dissection of the scar tissues, leading to a markedly increased prevalence of permanent morbidity following thyroid reoperations. In the present study, the risk of initial TT did not overweight the cumulative risk of postoperative and post-revision permanent morbidity of more limited thyroid resection modes. This evidence is in favor of initial TT for treatment of benign MNG. Nevertheless, recurrent goiter was described even after TT, the condition that is extremely rare (0.3%), and was usually caused by an inadequate resection of embryologic remnant thyroid tissue along the thyrothymic ligament or pyramidal tract [24]. Hence, thyroidectomy with much attention paid to identify and remove the entire thyroid gland along its embryologic descent should effectively eliminate benign goiter recurrence following TT [24].

The major strength of this study is that it is an RCT with a long-term follow-up of 10 years that allowed for an objective estimate of the treatment effects of utilizing different thyroid resection modes for patients with MNG. On the other hand, the study protocol has several limitations that should be taken into consideration. First, patients included into this study were recruited from the southern part of the Polish territory that historically has been classified as an iodine-deficient area and endemic goiter area according to the International Council for Control of Iodine Deficiency (ICCIDD) criteria. Hence, primary outcomes of this study may not be universally translated into other cohorts of patients with MNG, particularly living in iodine-sufficient areas of the world. However, we believe that in iodine-deficient areas both iodine prophylaxis and levothyroxine treatment are needed for efficient long-term prevention of goiter recurrence following other than total thyroidectomy [7]. In addition, all the operations in this study were performed by high-volume thyroid surgeons performing more than 200 thyroid operations annually each. Hence, the prevalence of surgical morbidity reported in this study cohort may not be achievable for low-volume thyroid surgeons. Adam et al. [5] reported recently that 81% of all thyroid surgeries in the USA were undertaken by low-volume surgeons and 51% of surgeons performed only 1 case per year. Thus, more limited thyroid resections for benign thyroid disease may still be a safe and effective alternative for low-volume surgeons, whereas TT should be reserved for high-volume thyroid surgeons. Last but not least, the novel surgical adjuncts having a potential for improving functional outcomes of thyroidectomy, such as IONM of the laryngeal nerves or near-infrared fluorescence technique for parathyroid imaging, were not utilized in this study for initial surgery. However, one can expect that the rising popularity of these adjuncts in the future can make safe TT at experienced hands even safer [25, 26]. Encouraging data were recently published by Wojtczak et al. [27] who found that experience with IONM led to an increase in RLN identification, a decrease of RLN injury, and an increase in the safe utilization of TT in non-monitored thyroid operations. Thus, if surgery is chosen as definitive treatment for MNG and the permanent complication rate can be kept below 2%, TT should be recommended, as it prevents recurrent nodular goiter and eliminates the risk of reoperation in the future.

## Conclusions

Total thyroidectomy performed by a high-volume thyroid surgeon can be regarded as the procedure of choice for patients with MNG, as it entails a significantly lower prevalence of recurrent goiter and need for revision thyroidectomy than other more limited thyroid resections. In addition, the risk of initial TT does not overweight the cumulative risk of postoperative and post-revision permanent morbidity of more limited thyroid resection modalities.
